# Domain general frontoparietal regions show modality-dependent coding of auditory and visual rules

**DOI:** 10.1162/IMAG.a.29

**Published:** 2025-06-16

**Authors:** Jade B. Jackson, Anina N. Rich, Denise Moerel, Lina Teichmann, John Duncan, Alex Woolgar

**Affiliations:** MRC Cognition and Brain Sciences Unit, University of Cambridge, Cambridge, United Kingdom; Macquarie Performance and Expertise Research Centre, & School of Psychological Sciences, Macquarie University, Sydney, Australia; The MARCS Institute for Brain, Behaviour and Development, Western Sydney University, Penrith, NSW, Australia; Laboratory of Brain and Cognition, National Institute of Mental Health, National Institutes of Health, Bethesda, MD, United States

**Keywords:** cognitive control, domain general, audiovisual, MVPA, stimulus-response rules, frontoparietal cortex

## Abstract

A defining feature of human cognition is our ability to respond flexibly to whatwe see and hear, changing how we respond depending on our current goals. Infact, we can rapidly associate almost any input stimulus with any arbitrarybehavioural response. This remarkable ability is thought to depend on afrontoparietal “multiple demand” circuit which is engaged by manytypes of cognitive demand and widely referred to as domain general. However, itis not clear how responses to multiple input modalities are structured withinthis system. Domain generality could be achieved by holding information in anabstract form that generalises over input modality, or in a modality-taggedform, which uses similar resources but produces unique codes to represent theinformation in each modality. We used a stimulus-response task, withconceptually identical rules in two sensory modalities (visual and auditory), todistinguish between these possibilities. Multivariate decoding of functionalmagnetic resonance imaging data showed that representations of visual andauditory rules recruited overlapping neural resources but were expressed inmodality-tagged non-generalisable neural codes. Our data suggest that thisfrontoparietal system may draw on the same or similar resources to solvemultiple tasks, but does not create modality-general representations of taskrules, even when those rules are conceptually identical between domains.

## 1 Introduction

Our sensory environment holds an abundance of information. This information is partlyprocessed in specialised neural structures whose architecture supports a particulardomain of processing, for example, primarily auditory input. However, flexible humancognition is generally thought to also require domain-general processing areas,capable of representing and integrating inputs from multiple modalities. Candidatedomain general areas of the brain have long been identified (Duncan & Owen, 2000;Fedorenko et al., 2013), but we donot know much about how information from multiple modalities is processed in theseregions. For example, it is not clear to what extent cells in domain general regionsexhibit preferences for sensory input from one modality over another, and to whatextent they can be fully re-allocated to code different types of sensoryinformation. Moreover, even if the neural resources are shared, it is not known towhat extent information is represented in a similar or different way for eachmodality. In principle, the same information, arising from two different modalities,could be represented abstractly, with shared underlying neural codes, orindependently, with non-generalisable patterns.

The frontal-parietal multiple-demand (MD) network is a functionally integrated neuralcircuit that is recruited by many types of cognitive demand (Assem et al., 2022;Duncan, 2010;Duncan & Owen, 2000;Fedorenko et al., 2013). It isthought to play a key role in cognitive control by integrating the relevantinformation from multiple more-specialised systems, as needed for the currentcognitive operation (Cole &Schneider, 2007;Cole et al.,2013;Cocuzza et al.,2020;Duncan et al.,2020). The regions of this system show high correlations of theirfunctional timeseries both with and in the absence of a cognitive task (Ji et al., 2019;Power et al., 2011), co-activateduring different demanding tasks including those associated with working memory,selective attention, and problem solving (Assem et al., 2020;Fedorenkoet al., 2013;Shashidhara etal., 2020), and dynamically adjust their system-wide connectivity betweentasks (Cole et al., 2013;Cocuzza et al., 2020). Single-unitwork in non-human primates has shown that information from different modalities isrepresented within this network, with prefrontal and parietal neurons encodinginformation about both auditory (Azuma& Suzuki, 1984;Romanski, 2007) and visual stimuli (Freedman & Assad, 2006;Freedman et al., 2001;Rao et al., 1997). Similarly in human brain studies, theMD network codes a variety of task features, including task-relevant informationfrom tactile (Woolgar & Zopf,2017), visual, auditory, rule, and motor domains (Pischedda et al., 2017;Schultz et al., 2022;Vaidya et al., 2021;Woolgar et al., 2016), consistentwith the idea of a domain general network.

Within the domain of vision, we know that MD responses are flexible. For example, therepresentation of visual and rule information in these regions adjusts according totask demands (Woolgar et al., 2011;Woolgar, Afshar, et al., 2015).Visual studies have also shown that these regions exhibit preferential coding forattended over unattended objects (e.g.,Woolgar, Williams, et al., 2015) and relevant over irrelevant dimensionsof visual objects (Jackson &Woolgar, 2018;Jackson et al.,2017). Individual MD voxels have even been shown to contribute tomultiple codes for different task-relevant visual features (Jackson & Woolgar, 2018). Theneural patterns in these MD regions appear to be important for behaviour (Robinson et al., 2022;Woolgar et al., 2019) and causal forflexible coding elsewhere in the network (Jackson, et al., 2021).

Although there is less work on MD coding of auditory stimuli, similar to the visualdomain, this network responds flexibly to inputs from this modality (Erhart et al., 2021;Kong et al., 2014;Schultz et al., 2022;Uluç et al., 2018). Forexample, regions of the MD network have been shown to code the task-relevant spatiallocation of auditory stimuli (Kong et al.,2014). More recently,Schultzet al. (2022)used MVPA to map logic, sensory (auditory and visual), andmotor rule representations finding that cognitive control networks, including theMDs, coded a larger number of rules in each domain (logic, sensory, motor) thanother brain regions. In addition, recent work that employed univariate analyses withhigh-precision functional magnetic resonance imaging (fMRI) reported almostidentical activation maps for hard compared to easy versions of auditory and visualtasks, even at the single-subject level (Assem et al., 2022). These studies suggest that resources in the MDnetwork can be flexibly allocated to represent relevant task information in both thevisual and auditory domain.

**Fig. 1.**
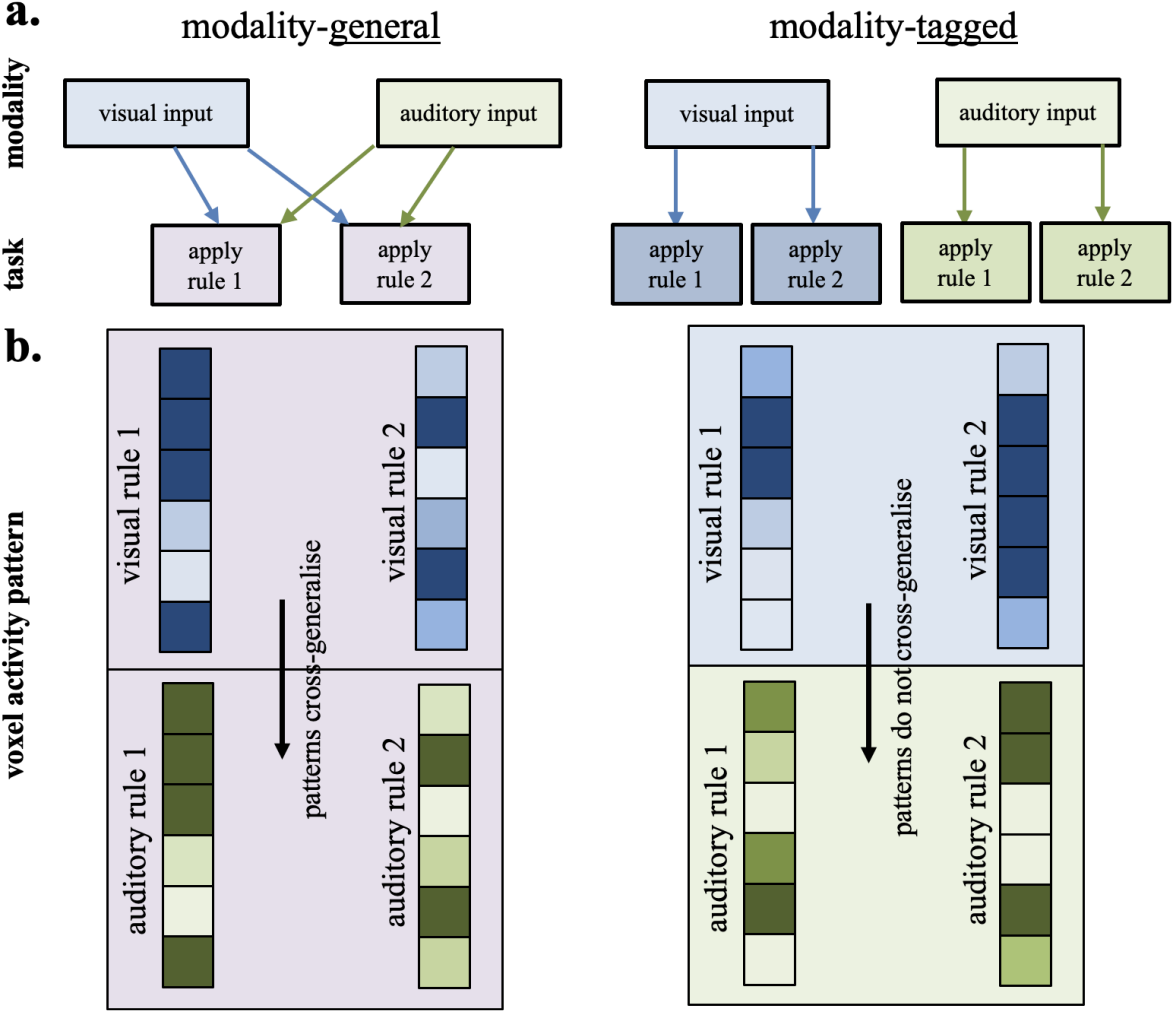
Prediction for modality-general vs. modality-tagged coding. This figuredepicts two possibilities for how information may be coded in a domaingeneral network following input from two different modalities. Panel (a)shows the modality inputs and the two task rules. Panel (b) shows an exampleof what the pattern representation would look like for each rule and whetherthis would cross-generalise. In the modality-general stream (left panel), wehave modality-independent representations of the rules in the visual andauditory tasks. Here, the same voxels are used in a similar way across thetwo modalities and the patterns are cross-generalisable. In the right panel(modality-tagged) we have an example of modality-dependent representationsof the rules in the visual and auditory tasks, where the same voxels in thenetwork may be used but in a unique way between the two modalities meaningthat the patterns do not cross-generalise.

Based on this prior literature, the MD network appears to be domain general, flexiblyrepresenting relevant rules and stimuli regardless of the source modality. However,there are different possibilities for how information from distinct modalities isencoded and spatially organised within these regions. This is important becausethere are several possible conceptualisations of domain generality. First, at themost basic level, a network could be considered domain general merely because itresponds to multiple inputs. This aligns with the original observation of the MDnetwork (Duncan & Owen,2000;Fedorenko et al.,2013), which is defined as a network that responds during differentcognitively challenging tasks. Such a result could arise if activity in theseregions reflected task-general processes such as effort or error monitoring, commonto many tasks. Second, a stronger requirement for domain generality might be thatthe network not only responds to, but also represents, multiple types ofinformation. For example, it could show specific patterns of activity from which itis possible to decode the details of, for example, both visual and auditory stimuli(Woolgar et al., 2016). Thiscould arise because the network comprises dedicated resources responding to eachmodality-specific task that are co-located in the same network or regions (Buschman, 2021), giving rise togenerality at the region or network, but not the single cell, level. Third, domaingenerality could be defined as when a network re-uses the*sameresources*to process these different types of information in differentsituations. This could arise if general-purpose resources are flexibly allocated toprocess information from each of the two modality-specific tasks (for example, as inBocincova et al., 2022;Manohar et al., 2019). This ischallenging to assess with fMRI (as even our highest resolution protocols capturethe activity of tens of thousands of neurons) but we can at least ask whetherpatterns from multiple modalities load onto the same or different voxels (Jackson & Woolgar, 2018).Finally, a network showing flexible re-use of general-purpose resources may expressthis domain generality in different ways. It could hold information in an abstractform that generalises over input modality (modality-general), or in amodality-tagged form, which re-uses the same resources but utilises unique codes torepresent the information in each modality (Fig. 1). In the former case, the same resources would be used in thesame way between modalities, and in the latter, the same resources may be used, butin different ways. Information about the input modality could be preserved in eitherscheme, in the former by inclusion of a separate modality-signalling code (anadditional dimension in the representational scheme (Badre et al., 2021)), and in the latter by changing thecontent code sufficiently between modalities so that codes do not generalise.

Here, we distinguished between these possibilities by considering how informationfrom two different sensory modalities is represented in the MD network. Participantsapplied identical stimulus-response mapping rules to visual or auditory stimuli.Using multivariate pattern analysis (MVPA) to characterise the MD representation ofthese rules, we asked 1) whether the MD network codes for rule information for bothauditory and visual tasks; 2) whether the same voxels contribute to both sets ofcodes; and 3) whether the codes underlying these rule representations aremodality-specific, or abstract and modality-independent. This allowed us to assesswhether visual and auditory task rules are represented in this network byindependent codes, or whether MD representation of task rules is abstracted awayfrom the input modality, with codes that generalise between auditory and visualtasks. Aligning with the concept of the MD system as a flexible resource, butagainst the intuition that conceptually identical rules should be represented in anabstract, generalisable form, our results show strongly decodable rules in the twomodalities that draw on overlapping resources but are represented in modality-taggednon-generalisable codes.

## 2 Methods

### 2.1 Participants

We recruited 49 volunteers for the experiment. Of these, 17 were excluded for notpassing the training or for not completing all the sessions (2 behaviouraltraining sessions plus 1 fMRI session). The final cohort therefore consisted of32 participants (mean age 24 years, SD = 4.1 years; 2 left-handed; 9 men,23 women). Participants were required to pass MRI safety screening, have normalor corrected-to-normal vision and no history of neurological or psychiatricdisorder. Participants were recruited through word of mouth and from theMacquarie University SONA participation pool at the Department of CognitiveScience, Macquarie University. They gave written informed consent to participateand were compensated for their time. The experiment was approved by theUniversity of Macquarie Research Ethics Committee (reference: 5201300541).

### 2.2 Task design

**Fig. 2.**
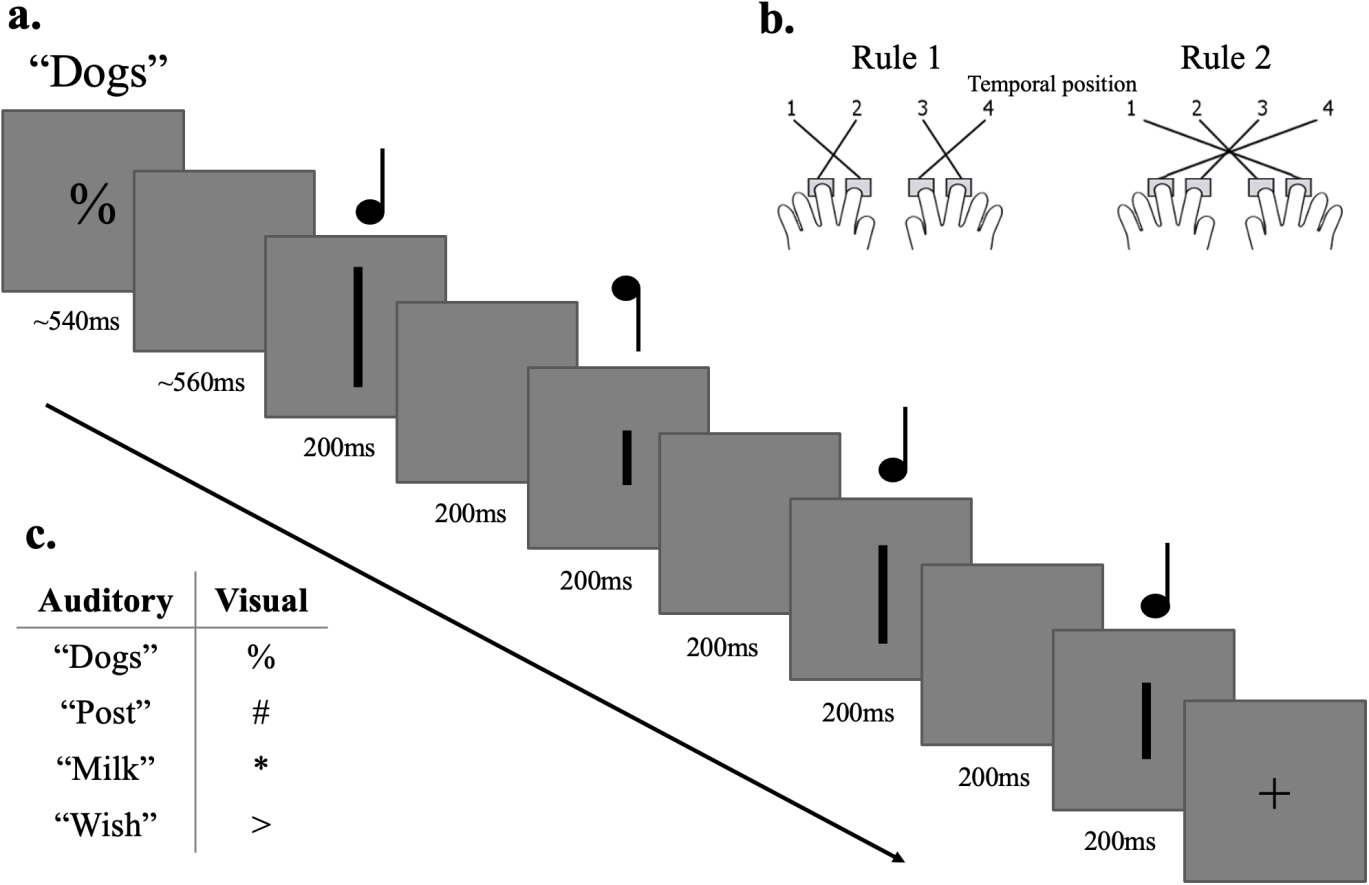
Task design. Panel (a) depicts a trial from the main task. Participantswere presented with a rule cue for ~540 ms. This was either a word(auditory task) or a symbol (visual task). In the auditory trials,participants were asked which tone had the highest pitch out of fourconsecutive tones. A black fixation cross was presented on the screenthrough the duration of the auditory trial (not depicted here). In thevisual trials, participants were asked which line was the shortest outof four consecutive vertical lines. Stimulus presentation was 200 mswith a 200 ms gap in between each presentation. Panel (b) shows the twostimulus-response mappings (rules). For example, under rule 1, toindicate that the highest tone or shortest line was in the secondtemporal position (as shown), the participant should press the first(far left) button. Under rule 2, they should instead press the thirdbutton. Each rule had two cues per modality. The spoken words used forthe rule cues are fromPetitet al. (2020). Panel (c) shows the cues for the auditory andvisual modalities, which we assigned to the two rules in a manner thatwas counterbalanced over participants.

Participants completed a task with visual or auditory stimuli (based onJiang & Kanwisher, 2003),with trials from the two modalities randomly interleaved (Fig. 2a). Before the start of each trial a tonewas played, and simultaneously a white cross was displayed on the screen (500ms) followed by a black cross (500 ms) to indicate the upcoming trial. At thestart of each trial, the trial type (auditory/visual) and currentstimulus-response mapping rule were indicated by a word (auditory trials) or asymbol (visual trials). The durations of the auditory cues were 537.7 ms (dogs),542.5 ms (milk), 539.6 ms (post), and 538. ms (wish). The duration of the visualcue presentation was jittered to match (pseudorandomly counterbalanced withinrun). Together the cue and post cue period had a duration of 1.1 s. In theauditory trials, participants then heard four consecutive tones (200 ms each,200 ms between tones) and had to identify which tone (1-4) had the highestpitch. A black fixation cross was displayed throughout the auditory trials. Inthe visual trials, participants observed 4 consecutive vertical lines (200 mseach, 200 ms between displays) and had to identify which was the shortest (1-4).The response time out was 11 s and there was 500 ms of jitter before the startof the next trial. Participants responded by pressing one of four responsebuttons using the index and middle fingers on both hands. There were twodifferent stimulus-response mapping rules (Fig. 2b). Each rule had two cues per modality(e.g., for auditory trials for a specific participant, two words“dogs/post” for rule 1, and two words “milk/wish”for rule 2; cue-rule association counterbalanced over participants, seeFig. 2c). This was to avoid theclassifiers using activity related to the specifics of the cue to distinguishthe rule conditions. There was an equal number of trial types in each run(modality, rule, cue, and target position; 32 trials, in total 6 runs and 192trials).

### 2.3 Procedure

#### 2.3.1 Overview

Participants completed three sessions in total. In the first two sessions(training, 1 h each), participants completed titration and training for themain task. During these sessions, task difficulty was equated for theauditory and visual stimuli using a staircase procedure (below), andparticipants practiced the different stimulus-response mappings (rules). Inthe final session, participants completed the main task in the scanner.Below, we outline the procedure in more detail.

#### 2.3.2 Session 1 and 2 (training)

The training in this first session had five separate stages. In the firststage, participants practised identifying the temporal position of thehighest tone or shortest line, without the stimulus-response mappings orassociated rule cues, separately for the two task modalities. Participantswere presented with a block of trials for each modality and responded bysaying number 1-4, depending on which tone had the highest pitch (auditory)or which line was the shortest (visual). The experimenter would then enterthe number reported. There were two blocks in total (20 trials).Participants did not respond using the keyboard to avoid them explicitlypracticing a stimulus-response mapping that would conflict with thestimulus-response mapping used in the scanning session. Participantsreceived feedback on every trial (fixation cross changed to green or red,500 ms) and there was no response time out. In all stages of the experiment,the starting modality was counterbalanced by participant number.

In the second stage of training, participants repeated the first stage butthis time with a staircase to equate task difficulty for the auditory andvisual stimuli. The staircase procedure is an adaptive method that adjuststhe difficulty of the task based on how participants have performed inprevious trials (Zhang et al.,2019). Here, we adjusted difficulty by manipulating differencesin line length and tone. We ran 2 (4-down, 1-up) staircases per modality (4staircases total). A 4-down, 1-up staircase means that the difficulty levelis increased if participants make four consecutive correct responses ordecreased if participants make one incorrect response. This staircaseprocedure was chosen to ensure high task accuracy. The 4 staircases wereblocked and each staircase ended after a maximum of 10 reversals. Thethreshold was calculated by averaging the threshold at the final reversalsfor each staircase (discarding the first 4 reversals if there was an evennumber of reversals, or discarding the first 3 if there was an odd number ofreversals). The 2 threshold estimates per modality were then averaged.Participants received feedback on every trial (fixation cross changed togreen or red, 500 ms) and there was no response time out.

In the third stage of training, participants were shown the stimulus-responsemappings and were required to enter responses themselves using the keyboard.Participants used the A, S, K, and L buttons on the keyboard to respondusing the same fingers as in the fMRI task. Each stimulus-response mappingwas introduced sequentially (e.g., rule 1 visual stimuli, rule 1 auditorystimuli, rule 2 visual stimuli, rule 2 auditory stimuli), and theparticipants learnt to associate the rule cues with each rule. The startingrule was selected pseudorandomly. Participants completed 4 blocks (12 trialsper block) and after the first 2 trials of each block they were shown areminder of the rule. Participants received feedback on every trial(fixation cross changed to green or red, 500 ms) and there was a responsetime out after 6 s.

In the fourth stage of training, participants practiced the task with the tworule cues combined and had to use the cues to differentiate which rule toapply. In this stage, participants completed 4 blocks (2 blocks permodality, 40 trials in total). The rule-cue mapping was kept the same foreach participant throughout all sessions of the experiment. In the finalfifth stage, the modalities were also interleaved, making the task verysimilar to that in the scanning session. Participants still receivedfeedback in this fifth stage, and they completed a minimum of 80 trials.

At the start of the second training session, participants were asked again tosign a behavioural consent form. They then practised the (stage 5) taskuntil the time for that session had ended. Participants needed to achieve an80% accuracy threshold in both the auditory and visual task to be able tocontinue to the fMRI session.

#### 2.3.3 Session 3 (MRI)

In session 3, participants filled out an MRI consent and screening form. Toremind them of the task, participants practised the task with feedbackoutside the scanner room (minimum of 80 trials). They then receivedinstructions on the button box, and we acquired a structural scan. To getfamiliar with the scanner setup and button box, participants practised thetask in the scanner for 8 trials in total (with feedback). Following this,they completed six runs of the main task without feedback (refer toSection 2.2).

### 2.4 Data acquisition

We collected the data with a Siemens (Erlangen, Germany) 3-T Verio scanner atMacquarie Medical Imaging, Macquarie University Hospital, Sydney, Australia. Weacquired a T1-weighted structural image for each participant (1 mm isotropicvoxels, repetition time (TR) 2000 ms, echo time (TE) 2.36 ms). To avoid acousticnoise from the scanner during presentation of the auditory (and visual) stimuli,we used an interleaved steady state (ISSS) imaging sequence (Peelle, 2014;Peelle et al., 2010), with adelay of 1 TR (RF and pulsed steady state silent mode, with 60us ramp time)after every 4 volumes. Volumes were acquired using interleavedT2*-weighted EPI acquisition with the following parameters: TR 3000 ms;TE 33 ms; 32 slices of 4.5 mm slice thickness with no interslice gap; in-planeresolution 3.0 × 3.0 mm; field of view 250 mm; and voxel size 3 x 3 x 4.5mm.

### 2.5 Preprocessing

We preprocessed the MRI data using SPM 12 (Wellcome Department of ImagingNeuroscience,www.fil.ion.ucl.ac.uk/spm) in MatLab 2018a. We converted functionalMRI data from DICOM to NIFTI format and spatially realigned to the firstfunctional scan. We did not perform slice-timing correction due to thenon-continuous acquisition (ISSS sequence) (as inPerrachione & Ghosh, 2013). We co-registeredstructural images to the mean EPI. We smoothed the EPIs in-plane (in the x andy, but not in the z direction) with a 4 mm FWHM Gaussian kernel. We also highpass filtered (128 s) the data. Finally, we normalised the structural scans tothe T1 template of SPM12 (Wellcome Department of Imaging Neuroscience, London,UK;www.fil.ion.ucl.ac.uk), using SPM12’s segment andnormalise routine. This was to derive the individual participant normalisationparameters needed for transformation of ROIs into native space and to normalisethe searchlight classification maps derived in native space.

### 2.6 ROIs

We took 13 frontal and parietal MD ROIs from the parcellated map provided byFedorenko et al. (2013;available online athttps://imaging.mrc-cbu.cam.ac.uk/imaging/MDsystem). This mapconsists of regions that show increased activation with task demands across arange of tasks. This definition of the MD network shares a high degree ofoverlap with the previous definition (Duncan & Owen, 2000) derived from meta-analytic data, that weused in previous work (Jackson& Woolgar, 2018;Jackson et al., 2017;Jackson et al., 2021;Woolgar, Afshar, et al., 2015;Woolgar, Williams, et al., 2015). MD ROIs comprisedleft and right anterior inferior frontal sulcus (aIFS; centre of mass (COM)= ±35 47 19, volume = 5.0 cm^3^), left and rightposterior inferior frontal sulcus (pIFS; COM ±40 32 27, 5.7cm^3^), left and right premotor cortex (PM; COM ±28−2 56, 9.0 cm^3^), left and right inferior frontal junction(IFJ; COM ±44 4 32, 10.1 cm^3^), left and right anteriorinsula/frontal operculum (AI/FO; COM ±34 19 2, 7.9 cm^3^), leftand right intraparietal sulcus (IPS; COM ±29 −56 46, 34.0cm^3^), and bilateral pre-supplementary motor area/anteriorcingulate cortex (pre-SMA/ACC; COM 0 15 46, 18.6 cm^3^). For the mainanalyses we combined these ROIs into one MD network ROI using FSL v5.08functions (Jenkinson et al.,2012). For decoding results from individual MD ROIs seeSupplementaryMaterials.

We defined early visual cortex (BA17: COM −1 −79 6, 31cm^3^) and primary motor cortex (BA4: COM ±27.8 −2360, 51.6 cm^3^) from the Brodmann template provided with MRICroN (Rorden, 2007). We also definedprimary auditory cortex (A1) as a spherical ROI (radius 10 mm) placed at theintersection of BA 41 and 42 within Heschl’s gyrus (COM ±50−22 10, 31.9 cm^3^). The decoding results for the visual andauditory ROIs can be found in theSupplementary Materials. We took the limbicnetwork from the 7 network parcellated map provided byYeo et al. (2011). These networks are derived fromresting-state functional connectivity.

All ROIs were deformed into native space by applying the inverse of thenormalisation parameters for each participant.

### 2.7 First-Level Model

To obtain activation patterns for MVPA, we estimated two separate GLMs for eachparticipant (SPM12). For the main model, we estimated the activity associatedwith the two visual, and two auditory rules, using correct trials only (fourregressors). To account for trial-by-trial variation in reaction time (Todd et al., 2013), trials weremodelled as events lasting from the offset of the fourth stimulus until responseconvolved with the hemodynamic response. We modelled this period as we reasonedthat, as this time window followed the four auditory or visual stimuli, thiswould be when participants made their decision and applied the rule, andprevious work has shown that there is a strong representation of the rulesduring the execution or recall phase (e.g.,Quentin et al., 2019).

We then ran a second model designed to capture button press responses. We usedthis as a common sense check for whether we could successfully decode the givenresponse (inner vs. outer finger position) in each task modality separately(within-modality decoding), and whether this information could becross-generalised across modality (between-modality decoding) in motor regions.For this we estimated the activity associated with the inner and outer fingerresponses across both hands, for the visual and auditory tasks separately (fourregressors total, modelled as events lasting from fourth stimulus offset untilresponse convolved with the hemodynamic response).

For both GLMs we included dummy scans and movement parameters (translation androtation) as covariates of no interest (totalling seven regressors). Wemaintained the correct relationship between events and fMRI volumes by addingdummy volumes into the time series to occupy the silent time periods where nodata were collected. As a reminder, the purpose of the silent time periods wasto avoid acoustic noise from the scanner during stimulus presentation (seeSection 2.4). We then removed theirinfluence on parameter estimation by perfectly modelling them with a singleregressor (0 for actual volumes, 1 for dummy volumes) that was not convolvedwith the haemodynamic response function (as described inPeelle, 2014). As we did notperform slice-timing correction (ISSS sequence), we also estimated temporalderivatives to account for slice-time differences. Temporal derivatives wereestimated for the main task (the visual and auditory rules in the first GLM, andthe inner and outer finger responses in the second GLM) and for the dummyregressors. We combined the two parameter estimates (e.g., visual rule 1, andits temporal derivative) by the root mean squared (Calhoun et al., 2004) and took these estimatesforward for our decoding analysis.

### 2.8 Analysis

We used MVPA to examine the representation of the rules applied to the visual andauditory stimuli. Of central interest was 1) whether the MD regions coded bothvisual and auditory-based rule information; 2) whether the same voxels werere-used for both sets of codes; and 3) whether rule representations generalisedacross modality, suggesting modality-independent rule representations.

We implemented MVPA using the Decoding Toolbox (Hebart et al., 2015). To address our first questionof whether the MD regions coded both visual and auditory rule information, wetrained the classifier to discriminate the two stimulus-response mappings(rules) for each modality separately (within-modality decoding). We did this inthe combined MD ROI, in each MD ROI separately, and in the visual and auditoryROIs. We also did this in the limbic network, as a null comparison model, withthe expectation that this network would not encode information about the twostimulus-response mappings.

For our second question, we took the voxels that contributed the most to ruleclassification in each modality (visual and auditory) and asked what proportionof these voxels was shared. For this we extracted the transformed classifierweights for each classification scheme (visual rule/auditory rule). Rawclassifier weights are not a simple reflection of the signal at each voxel, butan index of signal strength that can be recovered by multiplying the rawclassifier weights by the data covariance (transformed classifier weights,Haufe et al., 2014). Weidentified the voxels with the highest (top 10%) of these signal-reflectingtransformed classifier weights for visual rule coding and the top 10% of voxelswith the highest transformed weights for auditory rule coding, and asked howmany of these were the same voxels (expressed as a proportion, as inJackson & Woolgar, 2018).We then used a two-step permutation test (Stelzer et al., 2013) to test whether the proportionof voxels that contributed to both classification schemes in our data exceededthe proportion expected by chance. For each person, we trained a classifier onthe permuted condition labels for each classification scheme (visual rule1/visual rule 2, and auditory rule 1/auditory rule 2) which resulted in 32unique combinations (ways in which the conditions labels could be permutedacross all runs). We then built a voxel re-use null distribution for eachparticipant by randomly selecting one of the 32 unique combinations from eachclassification scheme and calculating the proportion of overlap in the top 10%of voxels with the highest transformed weights. We ran 10,000 permutations ofthis. We then built a group-level null distribution by sampling (withreplacement) 1000 samples per participant from these permutations and thenfinally averaging the 1000 samples across participants. From the group nulldistribution, we then calculated the probability of observing the actual voxelre-use value by means of the Monte-Carlo approach (*p*=*k*+ 1/(*n*+ 1)) where*k*is the number of permutations in the null with equal orhigher accuracy to the actual voxel re-use value and*n*is thenumber of all permutations. The estimate we used is slightly more conservativethan the unbiased estimate (*p=k / n)*and avoids returning a*p*value of 0(Hemerik & Goeman,2018;Phipson &Smyth, 2010). Separate to this analysis we also averaged thetransformed weights in each region of the MD network to depict the relativeweighting, or contribution, of individual MD ROIs to the two classificationschemes.

To address the third question of whether auditory and visual rule informationcross-generalised, we trained a classifier to distinguish the visual rules(visual rule 1 vs. visual rule 2) and tested performance on discriminating theauditory rules (auditory rule 1 vs. auditory rule 2), and*viceversa*, training on the auditory rules and testing on the visualrules (between-modality decoding). Next, as a positive control and to assesswhether we could distinguish between the codes representing the two modalities,we trained a classifier to discriminate between the auditory (rule 1 and rule 2data) and visual task (rule 1 and rule 2 data). We performed these analyses inthe combined MD network ROI, in each MD ROI separately, and in the visual andauditory ROIs. To check whether there were additional regions that showedcross-generalisation, we also performed an exploratory analysis in which wecarried out classification across the whole brain using a roaming searchlight(Kriegeskorte et al.,2006). For each participant, data were extracted from a spherical ROI(radius 5 mm) that was centred on each voxel in the brain. A classifier wastrained and tested using data from each sphere, and the classificationaccuracies were assigned to the central voxel. This yielded whole-brain accuracymaps for each individual. Accuracy maps were normalised and smoothed (8 mm FWHMGaussian kernel) for group-level analysis (one-sample*t*-test ateach voxel). The results were thresholded at*p*< 0.005with an extent threshold of 20 voxels.

As a common sense check, we assessed whether we could decode motor responses inthe visual and auditory-based tasks separately (within-modality decoding), andwhether these representations cross-generalised from the auditory to the visualtask and*vice versa*(between-modality decoding). For this wetrained the classifier to discriminate inner versus outer finger responses,training and testing within modality (visual/auditory), followed by training theclassifier on inner versus outer responses in the visual task and testing theclassifier on inner versus outer responses in the auditory task (and*vice versa*). We looked at information pertaining to thesemotor responses in the primary motor cortex (BA4).

Finally, we ran a cue cross-generalisation analysis to check whether ruledecoding was cue (in)dependent (combined MD ROI). The details and results ofthis analysis can be found in theSupplementary Materials.

**Fig. 3.**
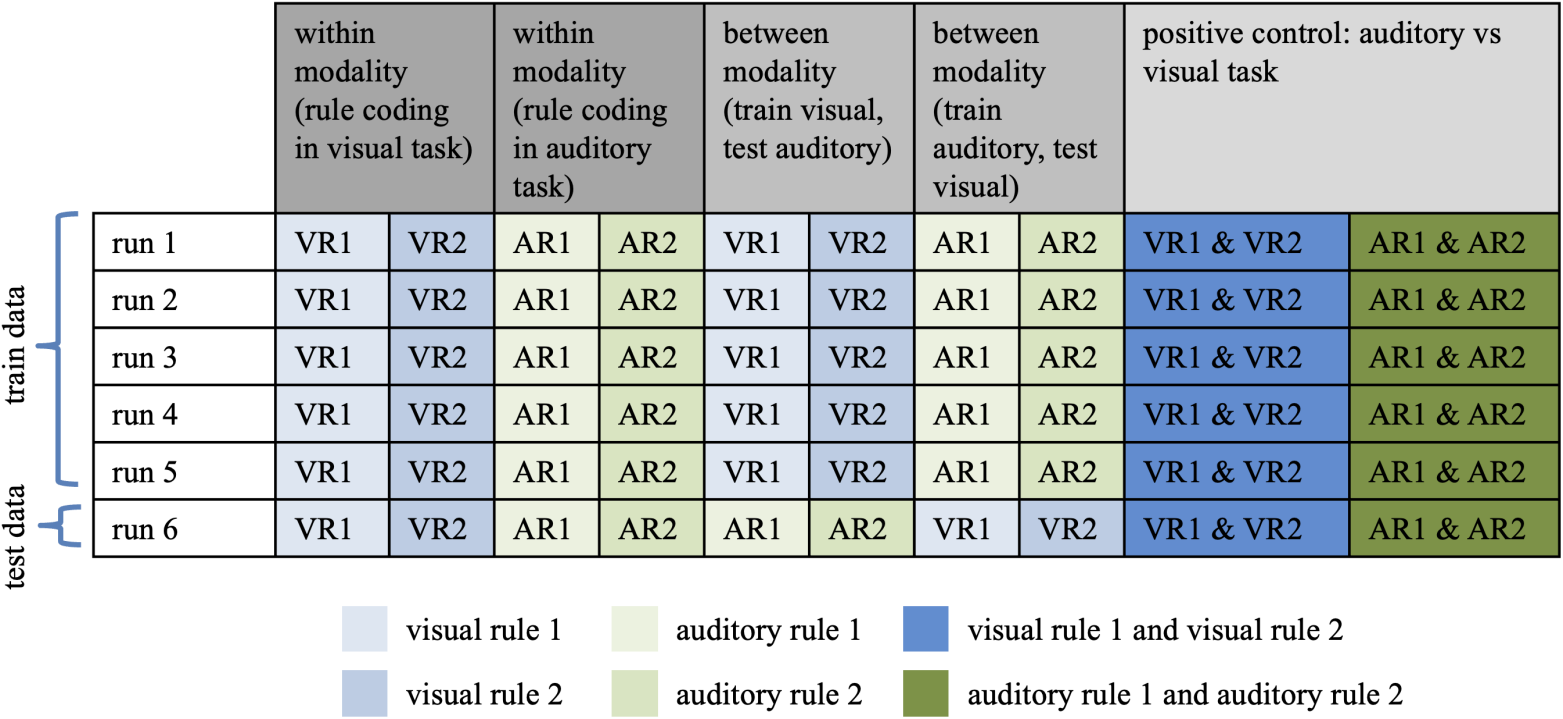
Classification schemes for within modality (rule coding in visual task,and auditory task), between modality (train on visual rules and test onauditory rules, and separately train on auditory rules test on visualrules), and the positive control (auditory vs. visual task). One exampleiteration is shown.

For all classification analyses we used a linear support vector machine and aleave-one-out six-fold splitter. For the within-modality decoding analysis, theclassifier was trained using the data from five of six runs (e.g., for thevisual modality: visual rule 1 vs. rule 2) and subsequently tested on itsaccuracy at classifying the unseen data from the remaining run (visual rule 1vs. rule 2), iterating over all possible combinations of training and testingruns. For the between-modality decoding analysis the classifier was trainedusing the data from five of six runs from one modality (e.g., visual rule 1 vs.rule 2, runs 1:5) and tested on its accuracy at classifying the left out runfrom the other modality (e.g., auditory rule 1 vs. rule 2, run 6), iteratingover all possibilities (and vice versa; train on auditory rules, test on visualrules). For our positive control to test if we could distinguish auditory fromvisual information, we again trained the classifier on five runs (visual rule 1and 2 vs. auditory rule 1 and 2) and tested on a final sixth run (visual rule 1and 2 vs. auditory rule 1 and 2) with all possible iterations.Figure 3depicts theseclassification schemes. The accuracies were averaged to give a mean accuracyscore. This was repeated for each condition, participant, and ROIseparately.

We used Bayesian statistics (Dienes,2011;Kass &Raftery, 1995;Morey etal., 2016;Rouder et al.,2009) to determine the evidence for above-chance decoding(alternative hypothesis) and chance decoding (null hypothesis) for eachclassification scheme and ROI using the Bayes Factor (BF) R package (Morey et al., 2016). We used ahalf-Cauchy prior for the alternative hypothesis to capture directional (abovechance) effects. The prior was centred around chance (δ = 0, i.e.,50% decoding accuracy), and had the default width of 0.707 (Jeffreys, 1998;Wetzels et al., 2011). Weexcluded the interval ranging δ = 0-0.5 from the prior todetermine very small effect sizes as irrelevant (Teichmann et al., 2021). We interpreted BFs below 1/3or above 3 as evidence for the null or the alternative hypothesis respectively(Wetzels et al., 2011). BFslarger than 3, or smaller than 1/3, are frequently taken as some evidencetowards or against the hypothesis, while values larger than 10, or smaller than1/10, are considered strong evidence (Jeffreys, 1998;Rouder etal., 2009). Importantly, BFs should be interpreted as continuous andaccordingly we report the actual Bayes Factor value obtained and depict theplots on a continuous scale rather than just the evidence threshold (Schmalz et al., 2023).

## 3 Results

### 3.1 Behavioural data

Reaction time and accuracy data from the scanning session are depicted inFigure 4. Participants performedwith a high degree of accuracy (mean percent correct for the visual rules= 93.6%,*SD*= 4.9, mean percent correct for theauditory rules = 94.7%,*SD*= 5.3). There wasevidence for no difference in accuracy scores between the two modalities at thegroup level (Bayesian paired t-test BF_10_= 0.3). There wasstrong evidence that participants took longer to respond on the auditory taskthan the visual task (mean auditory RT = 1699.3 ms, mean visual RT= 1165.6 ms, Bayesian paired t-test BF_10_= 391002.3).This was also the case for 28 out of 32 participants at the single subjectlevel.

**Fig. 4.**
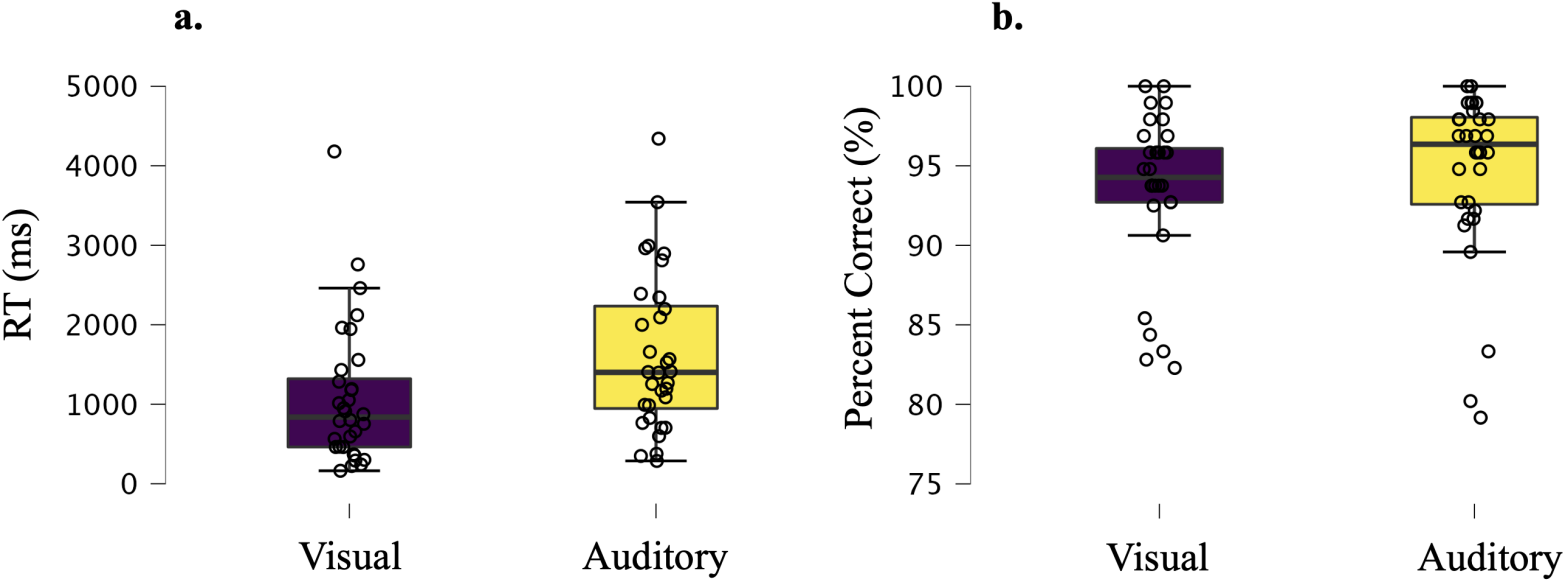
RT (correct trials only) and accuracy (percent correct, %) data. Boxplots(plotted in JASP (Team,2024)) with individual data points (open circles) in Panel(a) show RT (ms) data and Panel (b) show accuracy data. Boxplot innerline depicts median, and the box spans the range from the 25th to the75th percentile. The whiskers extend to the 25th percentile -(1.5* interquartile range) for the lower boundary, and 75thpercentile + (1.5* interquartile range) for the upperboundary. If the value for either is 0, then the whiskers extend to thedata extremes. Participants performed with a high degree of accuracyoverall due to the extensive training and performance criteria forprogressing to the fMRI session.

### 3.2 Coding of visual and auditory-based rule information

To address our first question of whether the MD network codes rule information inboth visual and auditory tasks, we used MVPA to differentiate the patternspertaining to the visual (visual rule 1 vs. visual rule 2) and the auditoryrules (auditory rule 1 vs. auditory rule 2) separately. The resultingclassification accuracies and associated BFs are shown inFigure 5a. Rule information was encoded in the MDnetwork (combined ROI) when participants completed the task in the visual (meanclassification accuracy 58.4%, BF_10_= 233.8) and the auditorydomains (mean classification accuracy 57.4%, BF_10_= 3.1). Forindividual MD ROI results refer toSupplementary Figure 1. As a comparison to theMD network, we also looked at whether the rule information in the visual and/orauditory tasks was encoded in the limbic network. There was evidence towards thenull that there was no coding of rules in the visual task in this network (meanclassification accuracy 50.7%, BF_10_= 0.19), and the BFsshowed inconclusive evidence for coding of rules in the auditory task (meanclassification accuracy 53.4%, BF_10_= 0.49).

**Fig. 5.**
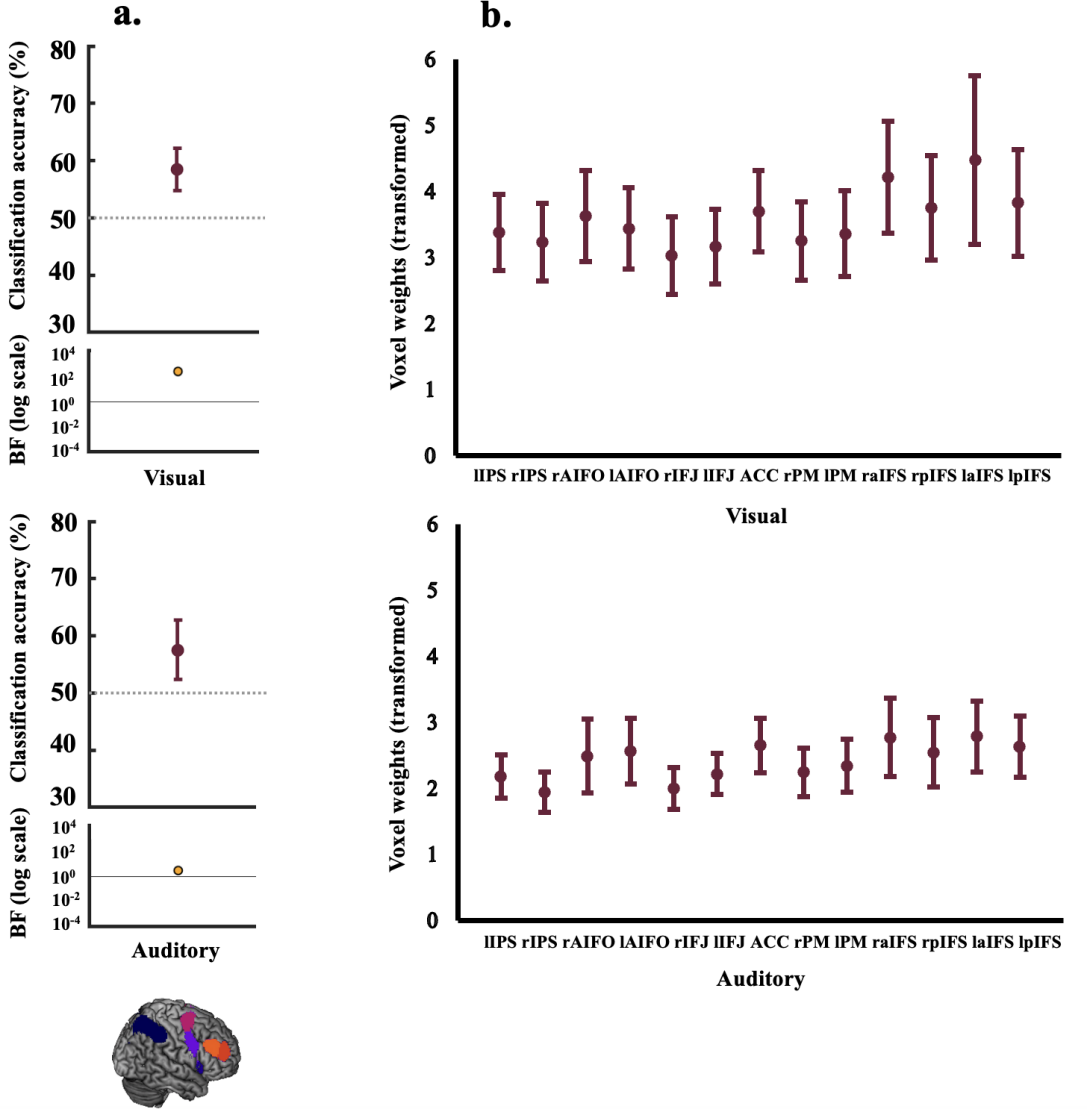
Decoding results and transformed weights for combined MD network ROI.Panel (a) shows coding (classification accuracy, %) of visual rules(upper) and auditory rules (lower) (within-modality decoding). The lowerparts of the plots show the associated BFs on a logarithmic scale,generated using custom code (fromTeichmann et al., 2021). BF_10_< 1/3 are marked in blue, and BF_10_> 3 aremarked in orange-coloured circles. There was evidence to indicate thatthe MD network encoded rule information in both the auditory and visualtasks. Panel (b) depicts a projection of the transformed weights(absolute) for each classification scheme (visual rules, auditory rules)averaged in the individual MD ROIs. These plots indicate thatclassification of the auditory and visual rules was not driven by anyregion of this network in particular. For plotting purposes, webootstrapped 95% confidence intervals across participants using 10,000bootstrap samples.

Next, we plotted the average of the transformed absolute classifier weights foreach classification scheme (visual/auditory;Fig. 5b) across the individual MD ROIs to assesstheir relative contributions. The weights were evenly distributed over ROIs,indicating that classification performance was not driven by any subset ofROIs.

### 3.3 Voxel re-use across auditory and visual tasks

Thus far, the results show that the MD network holds information pertaining toboth the auditory and visual rules. However, it is possible that informationabout these two modalities is still carried by independent populations ofneurons within this network. In this model, individual MD voxels might havedifferent preferences for rule coding in the two modalities. We examined theoverlap (re-use) in the 10% of voxels that contributed the most signal to eachclassification scheme and ran permutation tests allowing us to compare theobserved proportion of voxel re-use to that expected by chance. The MD networkdisplayed a higher proportion of voxels used in both classification schemes(33.4%,*p*< 0.0001) than what would be expected bychance (26.9%, group null average), suggesting that the same voxels were used toencode information about both task modalities.

### 3.4 Cross-generalisation of visual and auditory information

Our next question was whether the representation of rules cross-generalisedbetween modalities, that is, whether the MD network codes for rule informationin the same manner regardless of the input modality. Alternatively, it may bethat while overlapping voxels in this network encode information about bothmodalities, the codes are not shared and are independent. Consistent with thislatter possibility, the data presented inFigure 6ashow evidence for the null that therewas no cross-generalisation of auditory and visual rule information in thisnetwork. The mean classification accuracy for training on the visual rules andtesting on the auditory rules was 48.4%, (BF_10_= 0.16), andfor training on auditory and testing on visual, it was 51.1% (BF_10_= 0.11). Further, following a recent suggestion that cross-generalisationanalyses may be vulnerable if patterns are translated in representational space,for example due to a shift in mean activation between modalities (Taylor & Xu, 2024), wealso checked AUC output from our classifier. AUC should be robust totranslations as long as the structure of representational space remains thesame. There was still evidence towards the null effect of nocross-generalisation, with AUC for training on the visual rules and testing onthe auditory rules, 0.55 (BF_10_= 0.42), and for training onauditory and testing on visual, 0.52 (BF_10_= 0.11).

**Fig. 6.**
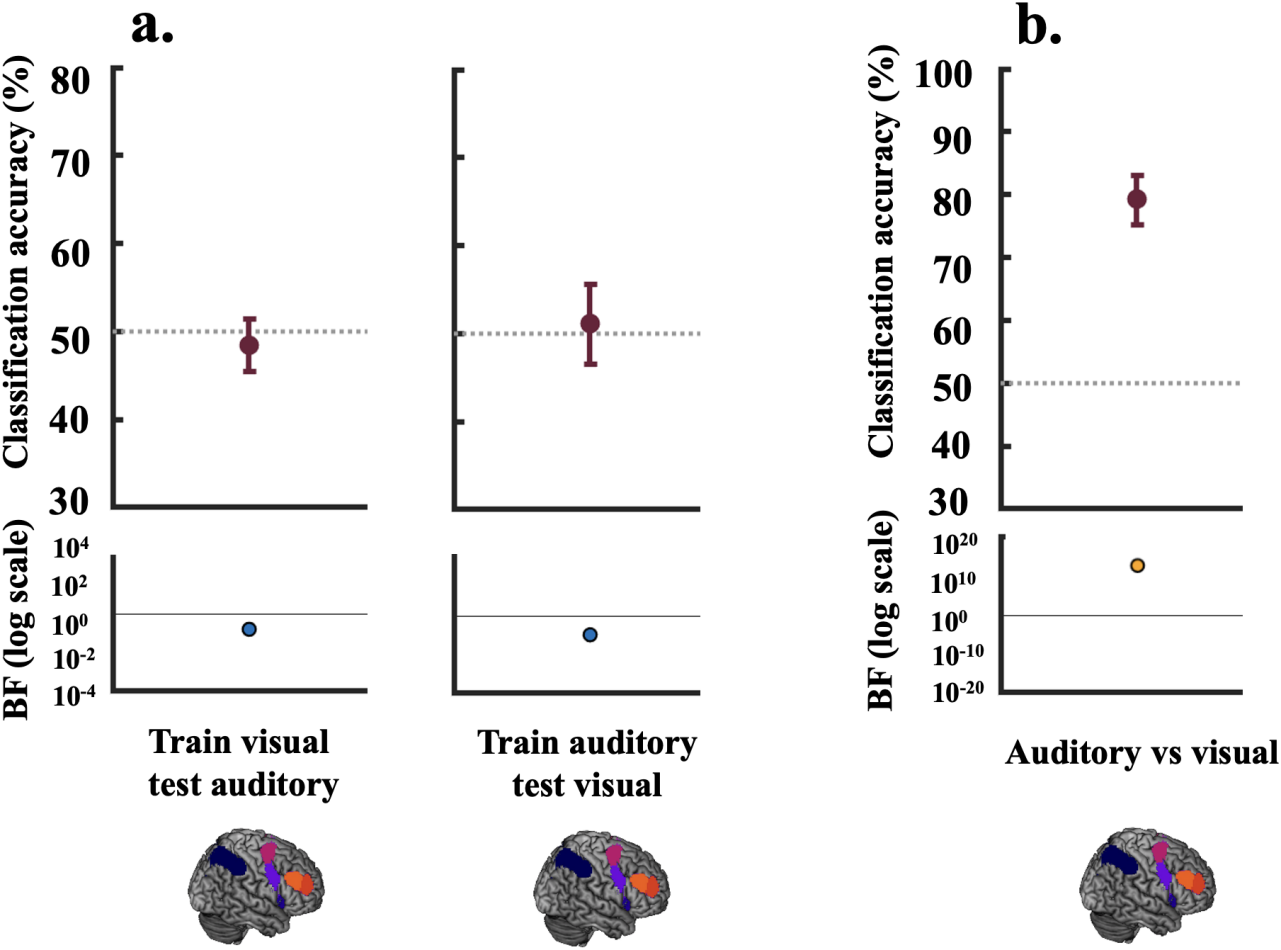
Decoding results for cross-generalisation (between modality decoding) vs.modality-specific representations. For Panel (a) the classifier wastrained on the visual rules (visual rule 1 vs. visual rule 2) and testedon the auditory rules (auditory rule 1 vs. auditory rule 2) (left panel)and vice versa (right panel). The lower parts of the plots show theassociated BFs on a logarithmic scale, generated using custom code (fromTeichmann et al.,2021). BF_10_< 1/3 are marked in blue, andBF_10_> 3 are marked in orange-coloured circles.The BFs for cross-generalisation indicate evidence for the null(BF_10_[0.16, 0.11]) that the MD network does not hold anabstract modality-independent representation of the rules withcross-generalisable underlying codes. Panel (b) shows classification ofthe auditory (rule 1 and rule 2) versus the visual task (rule 1 and rule2). This positive control shows evidence that this network distinguishesbetween information pertaining to the two modalities (BF_10_= 4.96*1012). For plotting purposes, we bootstrapped 95%confidence intervals across participants using 10,000 bootstrapsamples.

We then tested the complementary question of whether this network holdsmodality-distinguishing representations by training the classifier todistinguish auditory from visual information. There was strong evidence (Fig. 6b) that this networkexhibited differential activation for the auditory and visual tasks (meanclassification accuracy 79.3%, BF_10_=4.96*10^12^). The data for the individual MD ROIs (Supplementary Fig.2) show a similar pattern. To identify if there were any regionsshowing cross-generalisation outside of this network, we conducted anexploratory analysis using a roaming searchlight and assessed the results withcluster-level family wise error correction for multiple comparisons (voxelwisethreshold:*p*< 0.005 uncorrected). No clusters survivedthis correction.

Finally, we performed a common sense check for our cross-generalisationclassification analyses, assessing whether we could generalise motor responsesin BA4 across the auditory and visual tasks. We anticipated that we should notonly be able to successfully decode the given response (inner vs. outer fingerposition) in BA4 but moreover that this information would not be encodeddifferentially based on sensory modality (i.e., it would cross-generalise) andthis would therefore be a good test of the cross-generalisation analysis. Asdepicted inFigure 7, we wereable to decode the inner versus outer responses in both the visual and auditorytasks, and cross-generalise between them.

**Fig. 7.**
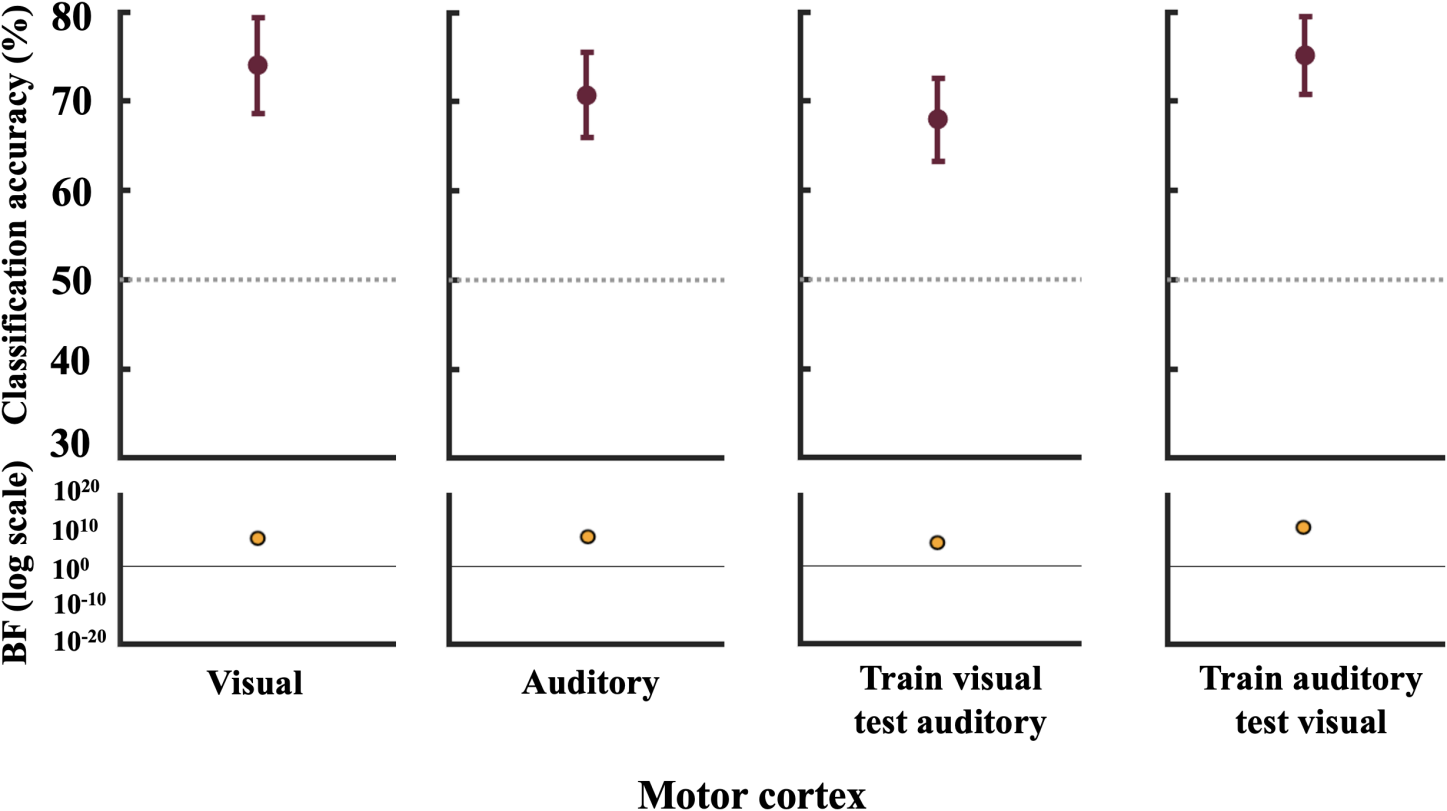
Decoding and BF results for the motor cortex. We performed a common sensecheck to assess whether we could decode motor responses and whether thiswas independent of modality. From left to right we decoded inner vs.outer finger responses 1) in the visual task, 2) in the auditory task,3) training in the visual, testing in the auditory, and 4) training inthe auditory, testing in the visual. For plotting purposes, webootstrapped 95% confidence intervals across participants using 10,000bootstrap samples. The lower parts of the plots show the associated BFson a logarithmic scale, generated using custom code (fromTeichmann et al., 2021).BF_10_< 1/3 are marked in blue, and BF_10_> 3 are marked in orange-coloured circles.

## 4 Discussion

In this study, we wanted to understand how domain generality arises in thefrontoparietal MD network, including whether information that could theoretically beabstracted away from the input modality would be coded in a modality-generalabstract form. We tested 1) whether the MD network of the human brain encodesinformation about both auditory and visual-based rules; 2) whether the same neuralresources (voxels) contribute to both sets of codes; and 3) whether the codesunderlying these task-relevant rule representations are shared across modality orremain modality-specific. There was evidence that this network coded ruleinformation for both auditory and visual tasks, corresponding with the dominant viewof this network as domain general. Further, more of the most strongly respondingvoxels than would be expected by chance contributed to both classification schemes,suggesting that some MD resources may be re-allocated to code task informationpertaining to different sensory inputs. However, there was evidence that thepatterns underlying coding of auditory and visual rules did not cross-generalise.Alongside this, there was evidence from our positive control that the classifiercould distinguish between the same rules applied to the two modalities. These datasuggest that although this domain general network holds information pertaining todistinct sensory inputs, and the same neural resources are implicated in doing so,the codes underlying the representation of this information are independent andtagged by modality, rather than shared and abstracted to the level of the rule.

The findings from this study complement ongoing research showing that the MD networkprocesses information from not only a wide range of tasks, but also acrossmodalities (Assem et al., 2022;Duncan, 2010;Duncan & Owen, 2000;Duncan et al., 2020;Schultz et al., 2022). The data alsofit well with the theory that this network is key to integration of various types ofinformation (Duncan et al., 2020).This is thought to be the case due to strong connectivity between the core regionsof the network (Assem et al., 2020)and task-dependent connections to other networks (Cocuzza et al., 2020;Cole & Schneider, 2007;Cole et al., 2013). Our data add to this base by showingthat not only are both visual and auditory rules encoded in these regions, but thepatterns for each tend to load onto the same MD voxels.

These results showed that, even with a presumably imperfect MD definition, morevoxels than would be expected by chance contributed to the representation of bothtypes of information (rule encoding in the auditory, and in the visual domain). Bycontrast, other work has shown modality biased subregions in and around the MDnetwork (Braga et al., 2017;Mayer et al., 2017;Michalka et al., 2015;Noyce et al., 2017). For example,contrasting visual and auditory attention tasks has revealed two visual-biasedregions along the superior and inferior precentral sulcus (PCS), interleaved withtwo auditory-biased regions along the transverse gyrus intersecting the PCS andalong the caudal portion of the IFS (Michalka et al., 2015;Noyceet al., 2017). Other MD regions (e.g., anterior cingulate and insula)have not shown sensory biases (Noyce etal., 2017) and using high-precision fMRI methods, sensory-biased areasidentified around lateral frontal cortex appear to lie adjacent to the MD borders(Assem et al., 2022). Takentogether, this previous work and the findings from the present study emphasise thatMD neurons may differ in their relative potential for coding different types ofinformation, and thus show sensory biases under specific circumstances, but thatthey are also highly adaptable (Duncan,2001) and can therefore potentially encode many different types ofinformation. What types of information are encoded may depend on many factors, forexample the nature of the task. Our data suggest that there is a flexible allocationof neural resources between our two tasks at least, perhaps reflective of mixedselectivity (Rigotti et al., 2013).Although it is important to note that while our voxel re-use analysis allows greaterspatial specificity than looking at whole regions, we cannot draw conclusions at theneural level as independent populations may underlie these voxel responses. Thecurrent picture suggests that while there may be sub-preferences towards differenttypes of information, MD cells are highly adaptable and can be flexibly allocated toencode different types of information, perhaps depending on the nature of thetask.

While some of the voxels with the strongest signal were the same across the twoclassification schemes, they did not appear to be used to form similar,modality-independent, codes. Instead, there was evidence for the null: there was nocross-generalisation between the auditory rules and visual rules, indicative ofindependent neural representations of modality-based rule information. By contrast,motor responses were decodable in the motor cortex, and it was possible tocross-generalise motor responses between the two modality-based tasks. In domaingeneral areas, independent representations of task rule for each modality wouldarise if general-processing resources randomly or systematically become associatedwith particular inputs and outputs, forming bespoke conjunctions. This aligns withrecent recurrent models of working memory in higher cortical regions (Bouchacourt & Buschman, 2019;Buschman, 2021;Manohar et al., 2019). In thesemodels, general-purpose “conjunction” units come to representconjunctions of stimuli due to fixed random recurrent connections (Buschman, 2021) or flexibly throughrapid Hebbian updating of synaptic weights that form bespoke conjunctions dependingon the current task (Manohar et al.,2019). Accordingly, neurons in these regions have been shown to respondto a conjunction of different sensory inputs, in different contexts and atparticular points in time (Aoi et al.,2020;Bocincova et al.,2022;Mante et al.,2013;Rigotti et al., 2013).These mixed-selectivity properties result in high-dimensional spaces forrepresenting cognitive variables and maintaining a unique combination of inputs,capturing the diversity of information from different brain regions (Badre et al., 2021;Bouchacourt & Buschman, 2019;Buschman, 2021). Onepossibility is that recurrent conjunctive coding in the MD system extends also tocombinations of stimuli and responses. Moreover, if this is underpinned by aflexible association process (Manohar etal., 2019), it could provide a basis for the human ability to respondflexibly according to current task rules. This would then align with the proposalthat a key role of the MD system may be to create the necessary associations betweenthe information and actions needed for the current task (Duncan et al., 2020). Our data fit well within thisframework, suggesting that the system flexibly responded here, using the sameresources to form independent representations of the stimulus-response rules in theauditory and visual tasks, rather than abstract, modality-independent, codes.

The choice of task design, imaging technique, and analysis will influence howinformation about different modalities is processed, represented, and read out. Forexample, inAssem et al. (2022)work, MD hard-minus-easy visual and auditory activation maps were close toidentical, suggesting that the MDs can respond to both types of information,comparable to the present data, but also suggestive that responses to controldemands in auditory and visual tasks may be similar. However, not only did the taskdesign differ (n-back working memory) to the present study, but so did the analysis,which was univariate and does not provide insight about the level of representation.Our multivariate analysis suggests that while domain general areas encodeinformation from both modalities, the information is represented in independentneural codes. In support of this, there was also strong evidence that the MD networkpatterns were distinguishable between auditory and visual information. This informsus that the network responds differently for auditory and visual trials. This resultcould have been driven by many factors, as the two tasks were well-matched but notidentical. For example, the two tasks necessarily comprised different stimuli, andit is possible that participants used different strategies in the two tasks, orfound one more effortful, as reflected in the longer reaction times in the auditorycondition. However, even if different strategies were employed it seems unlikelythat this would influence the mapping of stimulus to response which wastheoretically identical between modality and were the codes that we tested forcross-generalisation.

There are a few other details of the design that place limits on our interpretations.Our participant pool was predominantly female which may limit generalisation to thegeneral population. Further, sample size (in this study, n = 32) influencesthe proportion of times a BF results in the incorrect conclusion (Schmalz et al., 2023). For example,with an effect size of 0, default prior of 0.707, BF threshold of 3, and sample sizeof n = 30, the false positive rate is 7.5% (Schönbrodt et al., 2017). Moreover, our post-hocanalysis testing rule decoding cross-generalising from one cue set to another didnot show robust results, making it unclear to what extent cues influence thedecoding of rule (Supplementary Materials). This control analysis could be underpoweredand therefore should be interpreted with caution as there are only half the numberof trials in the training set on each side of the classifier compared to our mainanalysis. It seems unlikely that rule representation could be fully driven by thecue representations; however, as in addition to using two cues per modality, cuesoccurred a couple of seconds before the period of time that we modelled, withseveral stimulus events occurring in between. Finally, other work has specificallyconsidered rule representation during the encoding period (Pischedda et al., 2017;Reverberi et al., 2012), and these representations may bequite distinct from rule representations at execution (González-García et al., 2021). In our data,it would be difficult to fully separate activity associated with rule encoding fromexecution as we did not incorporate a long delay or jitter in between the cue periodand other tasks events. An interesting avenue for future research would be to lookat whether cross-generalisation of the rules can be observed during encoding.

Our cross-generalisation analysis appears to align with the idea that MD neurons canencode multiple task features (here, modality*rule) in a nonlinear,conjunctive fashion. However, we do not know the extent of this potentialnonlinearity, only that it is sufficient to prevent cross-generalisation of the fMRIdata. This leaves open the possibility that some MD cells do respond to differentmodalities in a more linear fashion, which our analysis was not sensitive to here.The percentage of neurons that exhibit mixed selectivity is unknown, and it may varyfrom one region to the next, and in any given task (Dang et al., 2022;Parthasarathy et al., 2017). We also cannot addresswhether non-generalisable coding is a general property of the MD system. It could beunique to specific features such as modality, while in other cases, task featuresare combined linearly (Shashidhara et al.,2024). For example, following training, monkey prefrontal neurons haveshown increased nonlinear mixed selectivity for task features in a spatial but notin a shape-based working memory task (Danget al., 2021). The way that task demands change over time may also impactthe level of abstraction at which representations are held. For example, again inmonkey prefrontal cortex (e.g.,Bernardi etal., 2020) it was shown that the level of abstraction at which varioustask representations are held changes before and during the course of a trial. Here,we used a low temporal resolution technique (fMRI) but future work could use neuralmeasurements with sufficient resolution, such as magnetoencephalography, to assesshow these types of representations change over time.

The MD network is widely thought of as domain general but there are many differentpossible conceptualisations of what domain generality is. Here, we sought tocharacterise domain generality in the MD system in terms of how information frommultiple sensory modalities is represented. Our findings confirm that the MD networkencodes information from multiple modalities, here rules pertaining to both visualand auditory tasks. As far as the resolution of fMRI permits, the data alsosuggested that similar neural resources were used to code for both sets of rules.However, the underling neural codes reflecting task rules in the context of eachmodality were not integrated to such a level of abstraction that we couldcross-generalise between them. We, therefore, suggest that while neural resources inthe MD system may be flexibly assigned to represent arbitrary conjunctions ofstimuli and responses, providing a basis for the human ability to respond flexiblyto what we see and hear, this ability does not rely on forming abstractrepresentations of rules that generalise over input modalities.

## Supplementary Material

Supplementary Material

## Data Availability

The ethical approval for this study does not allow us to share raw data openly.Source data for main reportFigures 5-7andSupplementaryFigures 1-3are available on Open Science Framework (OSF) (https://osf.io/hcpku). Template regionsof interest and the code used to analyse the current study are publicly available onOSF (https://osf.io/hcpku).
